# The Impact of Endoscopy Sedation Information Sheets on the Level of Concern Regarding Possible Awareness in Patients Undergoing Endoscopy Sedation

**DOI:** 10.3390/healthcare10010034

**Published:** 2021-12-24

**Authors:** Zi Ping Tong, Lincoln Gan Lim, Alison Pighills, Matthew Hiskens, Danny Bartlett

**Affiliations:** 1 Anaesthetics Department, Royal Brisbane and Women’s Hospital, Brisbane, QLD 4029, Australia; 2 Anaesthetics Department, Mackay Hospital and Health Service, Mackay, QLD 4740, Australia; lincoln.ganlim@health.qld.gov.au (L.G.L.); danny.bartlett@health.qld.gov.au (D.B.); 3 Mackay Institute of Research and Innovation, Mackay Hospital and Health Service, Mackay, QLD 4740, Australia; alison.pighills@health.qld.gov.au (A.P.); matthew.hiskens@health.qld.gov.au (M.H.)

**Keywords:** anaesthetics, awareness, conscious sedation, deep sedation, gastroscopy, colonoscopy

## Abstract

Sedation encompasses a continuum from complete unconsciousness to drowsiness and anxiolysis where some awareness might be expected. Most patients undergoing endoscopy sedation expect to be completely unconscious during the procedure and thus have unmet expectations regarding their state of consciousness. This study aimed to evaluate whether endoscopy sedation information sheets reduce the level of concern regarding possible awareness during endoscopy sedation at a major regional hospital. Our findings were that 28.8% of patients who received the endoscopy sedation information sheet (*n* = 82) were concerned about awareness during the procedure, compared to 36.5% of patients in the control group (*n* = 105). However, the difference was not statistically significant. We also found that the incidence of awareness was higher (13.9%) in the intervention group compared to 8.8% in the control group but, again, not statistically different. This study allowed us to elucidate the level of concern regarding possible awareness during sedation and the incidence of awareness during endoscopy sedation. This will enable future work investigating the role of endoscopy sedation information methods involving written and video material in assisting pre-procedure patient counselling.

## 1. Introduction

Awareness is a complication in patients undergoing general anaesthesia. However, sedation encompasses a continuum from complete unconsciousness to drowsiness and anxiolysis where some awareness might be expected. In moderate or light sedation, the objective is to minimise anxiety, pain and movement during endoscopy with the benefit of a faster recovery compared to deep sedation. Patients respond purposefully to verbal or tactile stimuli during moderate/light sedation. In Australia, most patients undergoing endoscopy sedation expect to be completely unconscious during the procedure. In a study done by Chatman et al. (2013), the majority of patients undergoing colonoscopy interpreted any awareness as a complication related to inadequate sedation/anaesthesia [1]. Patients were most concerned about the potential for procedural awareness, and only 22% of patients recognised that there was a chance of being aware during the colonoscopy. The patients who recognised the potential for procedural awareness reported significantly lower levels of concern regarding awareness than patients who were unaware of this possibility. A similar study found that patients undergoing sedation have unmet expectations regarding their state of consciousness and misinterpret the perception of any sensory stimuli beyond unconsciousness as unintended intraoperative awareness and recall [2]. Moreover, unexpected awareness during sedation can be associated with long-term psychological consequences [3,4]. As the use of sedation for procedures like endoscopy is common, the absolute number of patients who experience awareness will likely be noteworthy [5]. This highlights the importance of anaesthesia providers clearly setting appropriate expectations with preoperative information, as the explicit discussion of awareness reduces, rather than increases, patients’ level of concern [1].

At the Mackay Base Hospital, outpatient/elective endoscopy patients receive the Queensland Health “Upper Gastrointestinal Endoscopy” or “Colonoscopy” patient information sheets. (Table 1). The form “Upper Gastrointestinal Endoscopy” SW9165 v8 is available at www.health.qld.gov.au/consent. The form “Colonoscopy” SW9154 v8 was available for use by Queensland Health hospitals during the study period. The current form “Colonoscopy Consent (Adult)” SW9154 v11 is available at www.health.qld.gov.au/consent [6,7]. This consent information sheet provides details of sedation and the possible side effects. The information sheet briefly covers possible awareness in a sentence: “You may remember some or little about what has happened during the procedure”. More detailed information on procedural awareness is discussed in the “Anaesthesia for Endoscopy” information sheet published by the Australian Society of Anaesthetists (ASA): “Most patients wish to have little or no awareness or recall of the procedure. Please discuss your wishes with your anaesthetist before your endoscopy. Some awareness or recall can occur, as anaesthesia for endoscopy differs from anaesthesia given for surgical operations” (Table 2). The effectiveness of endoscopy sedation information sheets has been evaluated by several studies, with written and oral information rated as higher quality than oral information alone prior to endoscopy [8]. Existing literature also shows that colonoscopy patients prefer a combination of written information followed by a discussion of the information to check for understanding [9], and consent should be supported by easy-to-read information [10].

We hypothesised that, by providing patients with this endoscopy sedation information sheet via mail prior to the procedure, patients would have a more informed expectation of procedural sedation and, thus, have reduced levels of concern regarding procedural awareness. Patients would also be encouraged to raise any specific concerns or material risks regarding endoscopy sedation with their anaesthesia provider. This study allowed us to elucidate the level of concern regarding possible awareness during sedation and the incidence of awareness during endoscopy sedation.

## 2. Materials and Methods

Ethics approval was obtained from the Mackay Hospital and Health Service Research Governance Office (RGO) and the Townsville University Hospital Human Research Ethics Committee (HREC), reference HREC/16/QTHS/98. The clinical trial was registered with the Australian New Zealand Clinical Trials Registry (ANZCTR), registration number ACTRN12621001310853. Participants undergoing elective gastroscopy or colonoscopy for diagnostic purposes to rule out cancer at the Mackay Base Hospital were recruited over a period of 18 months. The exclusion criteria were: (1) inadequate English comprehension due to a language barrier, cognitive deficit or intellectual barrier, (2) English reading level below that of a grade 8 student, (3) patients aged less than 18 years, (4) significant cardiovascular or respiratory impairment (ASA IV and V) and (5) emergency and inpatient endoscopies.

### 2.1. Randomisation

Simple randomisation was used via computer-generated random numbers. Randomisation was carried out by a researcher who was not involved in recruiting participants or in delivering the study intervention. Once randomised, participants in the intervention group were sent the ASA “Anaesthesia for Endoscopy” information sheet about their forthcoming anaesthetic by a blinded research assistant. Participants were unblinded to the intervention as those in the intervention group received information sheets. Staff administering the questionnaires, anaesthetists and staff entering the data into the statistical software package (SPSS v19) were blind to group allocation.

### 2.2. Consent and Survey Information

The intervention group received both the Queensland Health “Consent Information” and the ASA “Anaesthesia for Endoscopy” information sheet while the control group received only the Queensland Health “Consent Information”. On the day of the endoscopy, the research coordinator enquired about whether the mailed endoscopy sedation information sheet was received before obtaining consent for participation in the study. Consenting participants completed a pre-procedure survey to gauge their level of concern regarding awareness, followed by a post-procedure survey to ascertain the occurrence of awareness, sedation adverse events (e.g., nausea and vomiting) and their satisfaction with their anaesthetic care. The research coordinator was experienced in methods of ascertaining patient eligibility for the study, and no patients were excluded based on the above exclusion criteria. The post-procedure surveys were completed in the post-anaesthetic unit or recovery room when participants became oriented to time, person and place.

### 2.3. Sedation and Endoscopy

Sedative drugs were administered according to the anaesthetists’ preferences, with no restrictions on total doses, methods of administration or drug combinations selected. All patients in the study received propofol-based IV sedation with or without other adjunct medication, such as short-acting opioids, midazolam and low-dose ketamine. Deep sedation was administered to both groups, as per common practice in Australia. Patients deemed unsuitable for deep sedation due to medical comorbidities and the very small minority of patients who requested for no sedation were excluded from the study. Deep sedation was assessed by the lack of purposeful movements or comprehensible verbal response during the entirety of the procedure. Drugs were titrated to maintain spontaneous ventilation throughout with minimal apnoea time.

### 2.4. Statistical Method

There is a lack of evidence to quantify the proportion of people likely to be concerned about awareness during endoscopy sedation; therefore, we assumed that roughly 75% of patients would be concerned about possible awareness. To show a 20% reduction in the level of concern (55% incidence of concern in the intervention group), we calculated that we would need to study 176 subjects to reject the null hypothesis, that there is no difference between the groups in the level of concern about possible awareness during sedation, with 80% power to detect a between-group difference at an alpha level of *p* ≤ 0.05.

Outcome measures are reported using both descriptive and inferential statistics. The primary outcome, level of concern regarding possible awareness during sedation, was analysed using the Chi-square non-parametric test. Significance tests were 2-sided at the 5% level for the primary and secondary outcomes.

Secondary outcome measures included: incidence of awareness during the procedure; level of knowledge of endoscopy sedation; incidence of adverse events (pain, respiratory complications, nausea, vomiting and excessive sleepiness); relationship between medication type, dose and adverse events; relationship between age, gender and adverse events; and satisfaction with both pre-endoscopy and overall anaesthetic care. Secondary outcomes which produced nominal or ordinal data were analysed as per the primary outcome measure. Secondary outcome measures which produced interval data were analysed using an analysis of co-variance (ANCOVA) for normally distributed data to enable adjustment for co-variates.

## 3. Results

A total of 187 participants were recruited, with 82 randomised to the intervention and 105 to the control group. Group characteristics are presented in Table 3 with group similarities in age, gender, bodyweight, procedure time, recovery time, propofol amount and previously undertaking a discussion about sedation with an anaesthetist. However, more people in the intervention group reported a previous endoscopy.

Table 4 presents the primary and secondary outcomes measures. 33.1% of all participants were concerned about being aware during the procedure, with the intervention group marginally less concerned about potential awareness (28.8% vs. 36.5%). However, this was not statistically significant (x^2^ 1.107 *p* = 0.33).

Overall, 10.8% of participants reported awareness, and the intervention group reported a greater incidence of awareness (13.9% vs. 8.8%). However, there was no statistically significant difference between the groups in recollection of events (x^2^ 0.602, *p* = 0.44).

The incidence of adverse events (nausea, vomiting, pain, excessive sleepiness and respiratory complications) was low at 6.9% of total participants. There was no statistically significant difference between groups in the occurrence of these events (6.3% of the intervention group and 7.7% of controls; x^2^ 0.103, *p* = 0.75). There was also no relationship between adverse events and participant age (Rho 0.1, *p* = 0.24); gender (Rho −0.004, *p* = 0.96); and weight (Rho 0.046, *p* = 0.59). There was no relationship between adverse events and dose of propofol (Rho 0.075, *p* = 0.42) or ketamine (Rho −0.058, *p* = 0.70).

A total of 98% of participants were satisfied with their pre-endoscopy anaesthetic care and the remaining 2% were neutral about it. A total of 99% of participants were satisfied with their overall anaesthetic care and the remaining 1% were dissatisfied. There were no statistically significant differences between groups for pre-endoscopy anaesthetic care (U 3088.5, *p* = 0.12) or overall care (U 3150, *p* = 0.26).

## 4. Discussion

This randomised controlled trial introduced an endoscopy sedation information sheet prior to the procedure as we hypothesised that this would increase patient knowledge and reduce concerns of procedural awareness. However, our study showed that the provision of information sheets did not result in a statistically significant increase in patients’ understanding of awareness during endoscopy sedation nor did it reduce their level of concern. This conflicts with findings from Garden et al. (1996), who provided information leaflets prior to obtaining patient consent and subsequently assessed knowledge of anaesthesia, including the possibility of waking during the procedure [11]. The leaflets had three levels of information: “full”, “standard” and “minimal” disclosure. Patients who received the most detailed set of information had significantly increased knowledge scores. Another randomised controlled study allocated patients to either a routine or a detailed information group, with information presented via an audio tape recording the day before the procedure [12]. The detailed information group was provided with additional details regarding anaesthesia complications, including awareness, and this resulted in a measurable increase in knowledge.

While these studies show that enhanced patient education improved knowledge of anaesthesia, they did not investigate changes in the level of concern regarding awareness during anaesthesia. Chatman et al. (2013) showed that patients who demonstrate understanding that awareness is a complication during sedation have a reduced concern regarding awareness [1]. However, we found no corresponding decrease in level of concern, and this is in line with a recent study of moderate/conscious sedation information sheets designed for education on comfort objectives rather than deep sedation, which found no improvement in patient understanding compared to verbal counselling alone [13]. These authors found that 59.8% of patients who received both written information and verbal counselling expressed understanding of moderate sedation compared with 68.6% of patients who only received verbal counselling (no statistical significance). The authors attributed these results to an inability to comprehend the written material (despite adequate English skills) and questioned if participants overreported reading the provided information. Based on these results, the authors recommended repetitive verbal counselling targeted to specific patient demographics (literacy level, culture).

Patients have varying knowledge levels of endoscopy sedation due to external sources of information, such as the internet, library, other patient information sheets, family or friends who have undergone the procedure, education level and having had a previous endoscopy. Our findings may be a result of these sources of information extraneous to the information sheet. A higher percentage of participants in the intervention group reported a previous endoscopy, and this could have made the intervention group more aware of what to expect with the anaesthetic and potentially less concerned about possible awareness. As mentioned, patients in both the control and intervention group received the Queensland Health “Consent Information–Local Anaesthetic and Sedation for your Procedure” which mentions procedural awareness, and it may be that this information is sufficient.

The same percentage of patients (55.1%) in both the intervention and control group indicated that they recalled discussing sedation with their anaesthetist. This discussion could have occurred during the pre-anaesthetic workup, which usually involves discussion of the anaesthetic technique. Participants who had a previous endoscopy could also have had a previous discussion about sedation. One might predict that, because more people in the intervention group had a previous endoscopy, more would have discussed sedation previously. Given our findings, we encourage the anaesthetist to discuss sedation with patients pre-procedure as a large proportion is not receiving this information.

Our study did not find a significant difference in the patients who could recall being aware during the procedure, with 10.8% of all participants reporting awareness. This is similar to the findings of Allen et al. (2015), where incidence of recall during elective outpatient colonoscopies was 12% for light and 1% for deep sedation [14]. Another paper by Stait et al. (2008) reported that the incidence of recall was 4% in a prospective observational study of 200 patients undergoing sedation for colonoscopy [15]. We found a trend toward greater incidence of awareness in the intervention group (13.9% vs. 8.8%), and this was possibly due to them receiving more information on the likelihood of potential awareness via the information sheet.

There were several limitations of this study, including that the number of patients recruited to each group may not have been sufficient to elicit a statistically significant response in outcome measures. This is due to the power analysis modelling we undertook, which assumed 75% of participants would be concerned about awareness, whereas the findings of our study indicated levels much lower than this. Analysis of our questionnaire identified the subjective nature of some of the prompts, such as self-assessing level of sedation knowledge before the procedure. Though this variety of interventional study introduces the potential of Hawthorne effect confounding, we controlled for this with the use of our control group. In a systematic review on anxiety associated with colonoscopies and flexible sigmoidoscopies, Yang et al. evaluated pre-procedure anxiety-lowering interventions [16]. Most interventions worked on improving the quality of information presented via video or written educational materials, with mixed results. It was suggested that information describing patient experiences, e.g., video testimonials, might be useful. A future direction can be to trial novel patient education methods.

## 5. Conclusions

This study found no difference in knowledge or concern regarding awareness during sedation for the group receiving the endoscopy sedation information sheet compared with control. This study allowed us to elucidate the level of concern regarding possible awareness during sedation and the incidence of awareness during endoscopy sedation. This will enable future work investigating the role of endoscopy sedation information methods involving written and video material in assisting pre-procedure patient counselling.

## Figures and Tables

**Table 1:** Queensland Health “Consent Information” information sheet—paragraph educating patients about sedation. Permission to publish obtained from Queensland Health.

**Will there be any discomfort? Is any anaesthetic needed?** The procedure can be uncomfortable and to make the procedure more comfortable a sedative injection or a light anaesthetic can be given. If you prefer, it can be done without sedation. Before the procedure begins the doctor: Will put a drip into a vein in your hand or forearm. This is where the sedation or anaesthetic is injected and May spray your throat with a numbing agent that will help prevent gagging.
**What is sedation?** Sedation is the use of drugs that give you a ‘sleepy-like’ feeling. It makes you feel very relaxed during a procedure that may be otherwise unpleasant or painful. You may remember some or little about what has occurred during the procedure. Anaesthesia is generally very safe but every anaesthetic has a risk of side effects and complications. Whilst these are usually temporary, some of them may cause long-term problems. The risk to you will depend on: Personal factors, such as whether you smoke or are overweight. Whether you have any other illness such as asthma, diabetes, heart disease, kidney disease, high blood pressure or other serious medical conditions

**Table 2:** ASA “Anaesthesia for Endoscopy” information sheet—section on possible adverse effects and potential awareness. Potential awareness during the procedure was highlighted by a heading and covered in more detail. Permission to publish obtained from Mi-tec Medical Publishing, Camberwell, VIC.

**Possible Risks and Complications** Anaesthesia for endoscopy is safe and effective. However, as with all medical and anaesthetic procedures, side effects are possible. Your anaesthetist is skilled at minimising these problems and managing them if they do occur. Common possible complications are: Pain in the arm on injection of the anaesthetic drugs Bruising after the intravenous cannula is removed Nausea and vomiting; the risk is less with endoscopies than with most other procedures Respiratory depression (decreased depth and rate of breathing) Fall in blood pressure (hypotension) Altered heart rate Dizziness or faintness, especially on starting to move around Rarely, an allergic reaction to the anaesthetic drugs (this can be life threatening in about one patient in 40,000) Vomitus from the stomach being inhaled into the lungs
**Awareness during the Procedure** As mentioned above, most patients wish to have little or no awareness or recall of the procedure. Please discuss your wishes with your anaesthetist before your endoscopy. Some awareness or recall can occur, as anaesthesia for endoscopy differs from the anaesthesia give for surgical operations. Your anaesthetist will always be with you and attending to your well-being.

**Table 3:** Group characteristics.

Variable	Intervention Group (*n* = 82)	Control Group (*n* = 105)
Age (yrs)	58 (13.45)	57 (14.24)
Gender (female, %)	48.8	56.2
Previous endoscopy (%)	61.5	46.9
Bodyweight (kg)	87.3 (22.11)	89.2 (21.57)
Procedure time (min)	28.03 (12.64)	28.15 (13.83)
Recovery time (min)	29.15 (10.14)	30.55 (11.93)
Propofol amount (mg)	322.57 (196.42)	337.21 (185.71)
Previous sedation discussion with anaesthetist (%)	55.1	55.1

Age, bodyweight, procedure time, recovery time, procedure time, propofol amount expressed as mean (SD).

**Table 4:** Results.

Outcome	Intervention Group (*n* = 82) %	Control Group (*n* = 105) %	Test Statistic and *p* Value
Pre-Procedure Survey			
Concerned about being aware of procedure (%)	28.8	36.5	x^2^ 1.107 *p* = 0.33
Level of sedation knowledge (informed/somewhat informed) (%)	89.2	82.1	U 2701.5, *p* = 0.12
Post-Procedure Survey			
Recall being aware during procedure (%)	13.9	8.8	x^2^ 0.602, *p* = 0.44
Incidence of adverse events (%)	6.3	7.7	x^2^ 0.103, *p* = 0.75
Satisfaction with pre-endoscopy anaesthetic care (%)	100	96.7	U 3088.5, *p* = 0.12
Satisfaction with overall anaesthetic care (%)	98.6	100	U 3150, *p* = 0.26

## Data Availability

Data supporting the reported results can be obtained by contacting the corresponding author.
